# Protective Role of Physical Activity and Antioxidant Systems During Spermatogenesis

**DOI:** 10.3390/biom15040478

**Published:** 2025-03-25

**Authors:** Cristina Antinozzi, Luigi Di Luigi, Laura Sireno, Daniela Caporossi, Ivan Dimauro, Paolo Sgrò

**Affiliations:** 1 Endocrinology Unit, Department of Movement, Human and Health Sciences, University of Rome Foro Italico, 00135 Rome, Italypaolo.sgro@uniroma4.it (P.S.); 2 Unit of Biology and Genetics of Movement, Department of Movement, Human and Health Sciences, University of Rome Foro Italico, Piazza Lauro De Bosis 15, 00135 Rome, Italy; l.sireno@studenti.uniroma4.it (L.S.); ivan.dimauro@uniroma4.it (I.D.)

**Keywords:** lifestyle, physical activity, sport, oxidative stress, spermatogenesis, steroidogenesis

## Abstract

Oxidative stress is a significant factor that contributes to male infertility and sperm dysfunction. In this condition, an increase in ROS production exceeds the body’s antioxidant defenses, resulting in a decline in spermatozoa quality and fertilizing capacity. Furthermore, excessive ROS production has been linked to the promotion of genomic damage, lipid peroxidation, inflammation, altered enzyme activity, and ultimately, irreversible alterations, cell death, and a decline in seminal parameters associated with male infertility. It is established that physical activity (PA), acting on inflammatory parameters and improving antioxidant defense, can alleviate the negative effects caused by free radicals, offering numerous health benefits and positively influencing sperm quality. The objective of this review is to highlight the mechanisms of ROS production, the physiological and pathophysiological roles of ROS in relation to the male reproductive system, and recent knowledge on the impact of some protocols of PA on these systems and the molecular mechanisms involved.

## 1. Introduction

Infertility is a significant global issue, affecting over 12% of couples worldwide, with male factors being, in approximately 20% of all cases, solely responsible, while in another 30% to 40% of all infertility cases, they are contributing factors [1,2]. Male fertility relies heavily on spermatogenesis, the process that produces large quantities of sperm cells (spermatozoa) in the testis. Spermatogenesis is a meticulously orchestrated biological process that culminates in the production of haploid spermatozoa capable of fertilizing an oocyte. This process involves three primary stages: proliferation of spermatogonia, the meiotic divisions, and the spermiogenesis [3]. One of the most significant aspects of the final stage is nuclear remodeling, where the compacting of DNA is achieved by replacing histones with protamines with the goal of safeguarding the genetic material, ensuring the optimal compaction of the paternal genomic material and its protection during the journey through the female reproductive tract [4]. Indeed, spermatozoa inherently generate large amounts of reactive oxygen species (ROS), impairing sperm motility and damaging DNA through the oxidation of membrane lipids and nucleic acids [5]. However, their role in spermatogenesis and sperm function is more nuanced, serving as both a potential disruptor and a key regulator of normal physiological processes. In spermatozoa, ROS mediate capacitation, hyperactivation and acrosome reaction, which are fundamental processes for fertilization, and they take part in intracellular signaling pathways essential for sperm development and function, such as the production of intracellular cAMP, which activates Protein Kinase A (PKA) [6]. Moreover, ROS contribute to cellular balance by regulating processes like vascular tone, oxygen sensing, immune response, and even adhesion properties.

This duality necessitates a robust but carefully regulated antioxidant system, and spermatozoa possess several intracellular antioxidant enzymes to mitigate the effects of ROS and the importance of antioxidants extends beyond endogenous mechanisms [7]. As a matter of fact, exogenous supplementation with compounds such as coenzyme Q10, L-acetyl-Carnitine, vitamin C, and zinc has been widely studied for its potential to counteract oxidative stress in semen. These supplements have demonstrated benefits in improving sperm quality and fertility outcomes, but excessive antioxidant use can lead to a condition known as reductive stress, where the over-suppression of ROS disrupts sperm functionality [8].

Physical activity (PA) is widely recognized for its numerous health benefits, including reduced risks of obesity, diabetes, and cardiovascular disease, pathologies in which oxidative stress-related inflammatory responses is considered a causative process [9]. Interestingly, it also appears to have a role in reducing oxidative stress in sperm, a key factor in maintaining male fertility. However, the relationship between PA and sperm quality is complex, with various levels and types of activity showing differing effects [10]. While moderate PA may provide protective benefits, strenuous exercise has been linked to reduced semen quality in some cases, particularly in activities such as long-distance running and cycling. Animal studies, for example, have shown that running can slow testicular aging by mitigating oxidative stress and it might be possible that regular exercise, improving the redox homeostasis, may lower the generation of ROS, thereby preventing damage to sperm DNA and cellular structures [11].

Indeed, the influence of PA in improving the antioxidant response and redox homeostasis is widely recognized [9], while the regular practice of physical exercise enhances semen quality by means of improving blood circulation and reducing systemic inflammation [10]. In the other hand, PA-induced antioxidant response, counteracting oxidative damage to sperm DNA and lipid membranes, may contribute to the improvements in sperm motility and viability [8].

This narrative review intends to offer an overview of the dual role of reactive oxygen species and antioxidant defense in spermatogenesis, focusing on the potential role of sport and physical activity in maintaining or impairing redox homeostasis control during spermatogenesis or in sperm.

## 2. Spermatogenesis

Spermatogenesis is the mechanism by which spermatozoa are produced from spermatogonial stem cells (SSCs). SSCs proliferate via mitosis to produce spermatogonia and then specialized haploid spermatozoa via meiosis, and spermiogenesis [12,13]. These cells migrate to the gonadal ridge and, after a period of mitotic division to form spermatogonia, enter meiosis. Meiotic divisions and cytological changes transform spermatogonia into spermatozoa, a highly specialized cell equipped with a tail that allows the cell to move. The head of the sperm contains the nucleus, which contains DNA and is located at the periphery, separated from the cell membrane and the acrosome, which contains various substances for penetrating the oocyte. The central part of the sperm contains the mitochondria, organized in different helical arrays around the axoneme. Mitochondria in spermatozoa are crucial for energy production, redox balance, calcium regulation, and apoptotic pathways, all necessary for sperm motility, capacitation, and fertilization [14]. Mitochondrial dysfunction and quantity can lead to decreased sperm quality and infertility [14].

Spermatogenesis is a complex process involving several hormonal and cells interactions. The hypothalamic release of gonadotropin-releasing hormone (GnRH) stimulates the anterior pituitary gland to secrete follicle-stimulating hormone (FSH) and luteinizing hormone (LH). Sertoli and Leydig cells are two major somatic cells that are pivotal for male reproduction [15]. These cells play critical roles in the process of spermatogenesis, ensuring proper development and maturation of sperm [16]. Sertoli cells, located within the seminiferous tubules of the testes, provide structural support and nourishment to developing germ cells [17]. They form the blood–testis barrier, regulate the environment of the seminiferous tubules, and secrete factors essential for the differentiation of spermatogonia into spermatozoa. Sertoli cells also produce inhibin, a hormone involved in the negative feedback regulation of FSH levels. Leydig cells, located in the interstitial space between the seminiferous tubules, influenced by LH, are responsible for the production of testosterone, which exerts its effects on Sertoli cells, promoting the maturation of germ cells and the progression of spermatogenesis. Consequently, the coordinated actions of GnRH, FSH, LH, Sertoli cells, Leydig cells, and testosterone work in concert to establish a regulated environment that facilitates the sustained production of sperm throughout the male reproductive life.

## 3. Redox Homeostasis and Physio-Pathological Conditions

**Figure 1:**
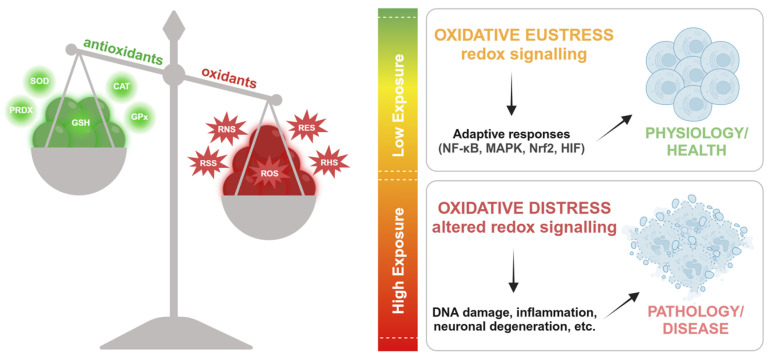
Redox balance and its physiological and pathological implications. The equilibrium between antioxidants (SOD, CAT, PRDX, GSH, GPx) and oxidants (RNS, RSS, ROS, RES, RHS) determines cellular redox responses. Low oxidant exposure induces *oxidative eustress*, promoting adaptive signaling (NF-κB, MAPK, Nrf2, HIF) and contributing to physiology and health. In contrast, high oxidant exposure leads to *oxidative distress*, disrupting redox signaling and resulting in pathological consequences such as DNA damage, inflammation, and neuronal degeneration. ROS—reactive oxygen species; RNS—reactive nitrogen species; RSS—reactive sulfur species; RES—reactive electrophile species; RHS—and reactive halogen species.

The concept of “oxidative stress” refers to an imbalance between oxidants and antioxidants, favoring oxidants, which results in disrupted redox signaling, impaired control mechanisms, and/or molecular damage [18]. Originally introduced in 1985 [19], this concept has evolved to encompass advancements in our understanding of redox signaling [20]. Fundamentally, it describes a steady-state redox balance within an open metabolic system, maintained at a specific setpoint that ensures basal redox tone. Any deviation from this balance constitutes stress, triggering a stress response. The definition also acknowledges that (i) a shift to the opposite end of the balance constitutes “reductive stress”, and (ii) deviations can be physiological (“oxidative eustress”) or supraphysiological (“oxidative distress”) [18] (Figure 1).

Oxidative eustress plays a crucial role in physiological redox signaling and control [18,19], aligning closely with the concept of redox homeostasis as the “golden mean” [21].

Metabolic regulation involves a wide array of chemical processes, including the orchestrated modification of proteins, lipids, carbohydrates, and nucleic acids to maintain structure and function. Redox reactions play a significant role in this regulation, with notable interactions between redox modifications and other regulatory mechanisms, such as the phosphorylation and dephosphorylation of proteins. Low-molecular-mass chemically reactive molecules, often referred to as “reactive species”, have been extensively studied for their regulatory roles. These include reactive oxygen species (ROS) [22], reactive nitrogen species (RNS) [23,24], reactive sulfur species (RSS) [25], reactive electrophile species (RES) [26], and reactive halogen species (RHS) [27]. The interplay among these reactive species forms a complex system of checks and balances essential for effective redox regulation [28]. On the opposite side of the redox equilibrium, the defense mechanisms against excessive oxidant levels involve a variety of antioxidant enzymes supported by their auxiliary systems, alongside low-molecular-weight antioxidants, collectively forming an integrated antioxidant network [29]. The expression of these antioxidant enzymes is regulated by key redox signaling pathways as part of the oxidative stress response [18].

Maintaining homeostasis is a fundamental aspect of health [30], which is regarded as a dynamic and active biological process [31]. Achieving and sustaining physiological health depends on mechanisms that ensure homeostasis through resistance, tolerance, and resilience, reflecting a homodynamic nature [32,33]. The concept of eustress, as distinct from non-physiological distress, characterizes the body’s continuous state of readiness to maintain homeostasis. This distinction, introduced by Selye [34], forms a foundational perspective in understanding stress and its responses [35].

Oxidation–reduction (redox) reactions are integral to life’s processes. Research into elements such as oxygen, iron, copper, sulfur, selenium, and nitrogen, alongside studies on free radical oxidation and defense mechanisms, has uncovered the remarkable versatility and applications of redox reactions. These include roles in energy capture, mitigating oxygen toxicity, and producing biochemical defenses against harmful entities. Notably, redox reactions also play a pivotal role in signaling, as they enable fast and reversible processes essential for physiological regulation.

The complex integration of energetically demanding systems makes life in oxygen-rich environments possible. Similarly, this principle applies to the regulation of redox signaling, which governs numerous physiological processes. Ensuring redox homeostasis presents an ongoing challenge, requiring tight biochemical control [18]. For oxidation events that serve as physiological signals, counteractive mechanisms must deactivate the signals and restore the redox balance to maintain health [21].

For over three decades, an understanding of ill health has been associated with the oxidative stress concept in the form of ‘an imbalance between oxidants and antioxidants in favor of the oxidants’, which developed into the idea of ‘disrupted redox signaling’ [36]. The refinement of this concept has been hastened by the articulation of a ‘Redox Code’ in an influential paper describing a set of principles through which biological function is enabled and protected [37]. Many disease processes are attributed to enhanced production of reactive oxygen species (ROS) or ‘dysfunctional Redox regulation’. Still, not everything can be explained by excessive ROS production. Many pathophysiological conditions may, in fact, exemplify processes that can be accounted for by other bioactive entities such as reactive nitrogen or sulfur species (RNS, RSS), or other small signaling molecules such as hydrogen (H2), ammonia (NH3), and carbon monoxide (CO). Many of these entities can react with each other, with protein thiols or other biomolecules.

These varied interactions modulate the function of ion channels, enzymes, transcription factors, and other biological targets, a scenario defined as the ‘reactive species interactome’ [38]. This reactive species interactome concept provides a useful framework to explain the apparent complexity of adaptive signaling. There is no single marker or process that captures the complexity of these interactions adequately. Rather, it is likely that a combination of readouts from different levels of organization will be required to explain how and why mitochondrial function appears to be so intimately related to chronic disease, inflammation and metabolism.

## 4. Redox Homeostasis and Spermatogenesis

Spermatogenesis is a highly regulated and continuous developmental process in which undifferentiated spermatogonia proliferate, undergo meiosis, and differentiate into mature spermatozoa [39,40]. This complex process is essential for male fertility and requires precise control to maintain testicular homeostasis. One of the key regulatory factors in spermatogenesis is redox homeostasis, which involves a delicate balance between reactive oxygen species (ROS) production and antioxidant defense mechanisms. Given the high metabolic activity required for sperm production, generating approximately 1000 sperm per second, mitochondrial oxidative phosphorylation is essential but also results in the production of significant levels of ROS [41,42]. Proper regulation of ROS is crucial, as excessive oxidative stress can impair spermatogenesis and male fertility [43].

ROS, including superoxide anions, hydrogen peroxide, and hydroxyl radicals, are natural byproducts of cellular metabolism, particularly mitochondrial ATP production. While low-to-moderate levels of ROS play physiological roles in cell signaling, differentiation, and sperm maturation [44,45], excessive ROS can be detrimental [43,46]. High levels of oxidative stress can lead to lipid peroxidation, DNA damage, and protein oxidation, ultimately disrupting the process of spermatogenesis [46]. An imbalance in testicular redox homeostasis has been associated with reduced sperm count, decreased motility, increased morphological abnormalities, and impaired fertilization capacity [46].

To counteract oxidative stress, cells involved in spermatogenesis are equipped with an array of antioxidant defense systems. Key antioxidant enzymes include superoxide dismutases (SODs), which convert superoxide radicals into hydrogen peroxide; glutathione peroxidases (GPXs) and peroxiredoxins (PRXs), which neutralize hydrogen peroxide; and glutathione S-transferases (GSTs) and thioredoxins (TRXs), which help maintain redox balance. These enzymes are widely expressed in testicular tissue and are regulated by redox-sensitive transcription factors, ensuring that oxidative stress does not reach detrimental levels [47,48].

Recent studies have also identified LanCL2 as a critical antioxidant gene specifically expressed in male germ cells, with high enrichment in spermatocytes and round spermatids [49]. Research using LanCL2 knockout mice has demonstrated that the absence of this gene results in testicular redox imbalance, leading to defects in spermatogenesis. LanCL2 deletion has been linked to premature spermatogonial self-renewal and impaired acrosomal maturation in spermiogenesis, ultimately reducing sperm count and motility. The disruption of testicular homeostasis in LanCL2-deficient mice suggests that this gene plays a crucial role in maintaining redox equilibrium and ensuring proper sperm development [49].

Genetic variations in antioxidant enzymes have been implicated in male infertility. Several polymorphisms in genes encoding key antioxidant proteins have been linked to compromised sperm function and increased susceptibility to oxidative stress [50,51,52,53,54]. In animal models, the deletion of specific antioxidant genes has resulted in heightened sensitivity to oxidative stress and age-related reproductive decline [55,56]. However, the absence of major fertility defects in most antioxidant-deficient male mice suggests that testis-specific oxidative defense mechanisms have yet to be fully understood. Identifying these unique redox regulatory pathways, including the role of LanCL2, could provide new insights into male reproductive health and potential therapeutic strategies for male infertility.

Redox homeostasis plays a fundamental role in regulating spermatogenesis by balancing ROS production and antioxidant defenses. While physiological levels of ROS are necessary for sperm development and function, excessive oxidative stress can disrupt spermatogenesis and lead to male infertility. The identification of testis-specific oxidative defense mechanisms, such as the LanCL2-mediated pathway, remains an important area of research, with potential implications for understanding and treating male reproductive disorders. Future studies focusing on redox regulation in testicular cells may pave the way for novel therapeutic interventions aimed at preserving male fertility.

## 5. The Role of Oxidative Stress in Male Infertility

**Figure 2:**
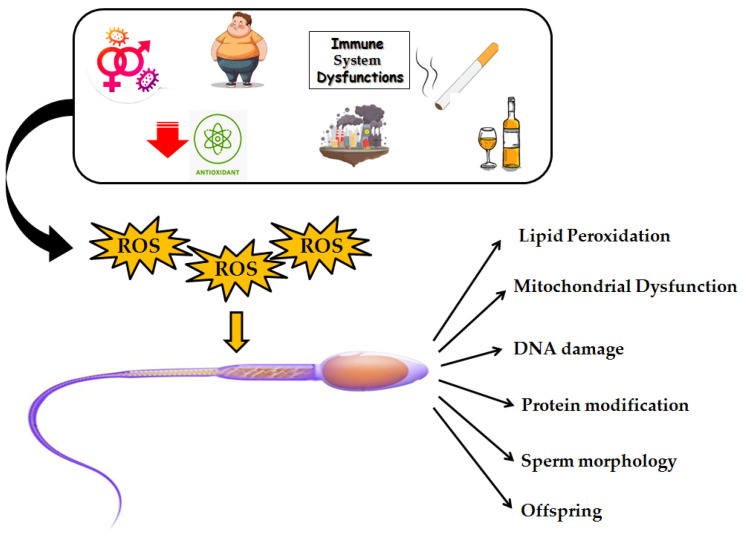
Endogenous and exogenous factors that alter oxidant/antioxidant balance, and that lead to oxidative stress and sperm dysfunctions.

Several conditions may possibly alter oxidant/antioxidant balance, which could in turn lead to oxidative stress. These conditions include endogenous factors such as deficiencies in antioxidants, immune system dysfunctions bacterial/viral infections, abnormal spermatozoa, leukocytospermia (LCS) [57,58], and exogenous factors such as smoke, pollution, alcohol, obesity, varicocele or sexually transmitted diseases [59,60,61] (Figure 2).

Oxidative stress induced by environmental pollution is a critical environmental issue that exerts a significant influence on male fertility [62]. Pollutants, particularly particulate matter (PM), nitrogen dioxide (NO2), and ozone (O3), generate ROS within the body, resulting in cellular damage. These ROS have been shown to overcome the body’s antioxidant defense mechanisms, contributing to inflammation, DNA damage and cellular dysfunction [63]. Several pollutants have been shown to induce oxidative stress in the male reproductive system [64]. Specifically, endocrine-disrupting chemicals, such as phthalates and bisphenol A (BPA), can mimic or interfere with the action of hormones, thereby disrupting the normal functioning of the reproductive system, impairing sperm development, and decreasing testicular size [65]. These detrimental effects are not limited to immediate consequences but can also have long-term implications for male fertility. In fact, studies have demonstrated that individuals exposed to high levels of environmental pollutants may experience a decline in sperm count and concentration over time [66]. Moreover, the impact of pollution on fertility can extend across generations, as oxidative damage to sperm DNA may result in epigenetic modifications, a DNA fragmentation index, and mitochondrial dysfunction, all of which can lead to male infertility [67].

Indeed, sperm plays a crucial role in intergenerational and transgenerational inheritance, particularly through epigenetic mechanisms [68]. Unlike genetic mutations, epigenetic changes involve modifications, such as DNA methylation, histone modifications, and small non-coding RNAs (ncRNAs) expression, that alter gene expression without changing the underlying DNA sequence [69]. The epigenetic marks respond dynamically to environmental and lifestyle factors, and when modified, they can transmit altered gene expression patterns to the offspring, influencing their health and phenotype [70,71]. Oxidative stress has been significantly implicated in transgenerational sperm effects [72]. As indicated above, mature sperm lack robust DNA repair capabilities due to chromatin compaction, so they are particularly vulnerable to ROS-induced damage [73,74,75]. Several situations may lead to sperm exposure to ROS, whether it is secondary to aging, environmental exposure, pathological situations, or lifestyle factors, such as unbalanced diet, smoking, alcohol addiction, or physical inactivity [4]. Antioxidant supplementation has been shown to improve sperm function, reduce DNA fragmentation, and maintain sperm motility and fertility, potentially mitigating transgenerational negative impacts [72]. Modification of sperm epigenome by paternal exercise before conception has been demonstrated, significantly impacting metabolic changes in the offspring. Several results indicate that paternal exercise can modify fetal development, placenta inflammation, and tissue-specific gene and protein expression patterns in offspring organs such as the heart, skeletal muscle, tendon, hippocampus, and liver [76]. Although exercise interventions in fathers have shown promising potential to positively shape offspring phenotypes, a notable limitation on this topic is the lack of compelling in vivo evidence substantiating a causal link between paternal oxidative stress and transgenerational epigenetic modifications that impact male fertility in humans, so that the precise biological pathways remain unclear [77].

Furthermore, the testicular environment is particularly vulnerable to oxidative damage due to the high metabolic activity of spermatogenic cells [78] and poor vascularization, as well as the presence of a high amount of polyunsaturated fatty acids (PUFAs) in the plasma membrane in conjunction with the absence of cytoplasmic antioxidant enzymes [79,80,81,82,83]. ROS, indeed, can potentially damage sperm membranes organization, mitochondrial activity, and the function of testicular cells [83], consequently affecting germ cells motility and ability to fuse with oocytes, testis steroid hormone synthesis, and steroidogenic capacity [79,84,85]. Mitochondria are the energy powerhouses of the sperm cell, and any disruption in their function can lead to diminished motility, thus reducing the sperm’s ability to fertilize an egg [86]. Studies have shown that men with lower sperm motility exhibit decreased glucose-6-phosphate dehydrogenase activity and increased levels of malondialdehyde, a marker of oxidative damage [78]. Furthermore, oxidative stress has been implicated in the disruption of the blood–testis barrier, the key structure that protects the developing germ cells from harmful substances, including ROS [87]. For these reasons, these cells are considered ’ideal’ bioindicators of pollution and early sentinels of human health [88].

Interestingly, it has been demonstrated that excessive intake of antioxidants, particularly through high-dose supplements, has been subject to concerns regarding its impact on male fertility [89]. Studies have shown that over-scavenging of ROS can impair essential sperm processes, such as capacitation and the acrosome reaction, reducing fertilization potential [77,90]. Additionally, excessive antioxidants may disrupt sperm maturation and lower sperm quality and motility, as well as interfering with testosterone production, ultimately decreasing sperm production [90]. Consequently, achieving a balanced intake of antioxidants through dietary means rather than relying on supplements is imperative to maintain optimal male fertility. However, all these factors are modifiable and reversible, and hence, by merely changing one’s lifestyle, many of these risk factors can be avoided.

### Testicular Cells and Oxidative Stress

The testis is composed of several different cell types, each playing a vital role in the process of sperm production. Sertoli cells, Leydig cells, and germ cells are all integral components of this dynamic environment, and each are influenced by the redox status of the tissue. Sertoli cells, which provide nutritional and structural support to developing germ cells, are particularly sensitive to changes in oxidative balance. Under conditions of chronic oxidative stress, Sertoli cell function is compromised, leading to impaired spermatogenesis and, consequently, a reduction in sperm count and quality [91]. The presence of ROS was associated with significant levels of apoptosis in Sertoli cells; a substantial decrease in connexin-43 (Cx43) expression, a key component of gap junctions, which is pivotal to spermatogenesis regulation; and a failure to maintain the viability of spermatogonial stem cells (SSCs) [92].

Research on Sertoli cells and oxidative stress suggests that various environmental toxins have the potential to induce oxidative damage and apoptosis. It has been suggested that exposure to 2,3,7,8-tetrachlorodibenzo-p-dioxin (TCDD) may possibly impair Sertoli cell function through oxidative stress, affecting mitochondrial activity and membrane potential [93]. Similarly, it appears that sodium fluoride exposure may contribute to a decline in cell viability and an increase in oxidative stress and apoptosis. Furthermore, acrylamide and its metabolite glycidamide have been observed to cause oxidative stress and apoptosis in both Leydig and Sertoli cells, affecting cell viability and gene expression of apoptotic markers [94].

Exposure to oxidative stress has been demonstrated to result in a decline in testosterone production, an escalation in apoptosis, and an impairment in steroidogenesis in Leydig cells [94,95]. In 2016, Duan et al. investigated the effects of hydrogen peroxide (H_2_O_2_) on primary rat Leydig cells and the role of peroxiredoxin 2 (Prdx2), an important antioxidant protein involved in oxidative stress response. The study revealed that H_2_O_2_ treatment led to a significant decline in cell viability, inducing apoptosis in a dose-dependent manner and altering Prdx2 protein expression [95]. Subsequently, in 2021, Anak and colleagues explored the impact of aging on testosterone production, conducting a comprehensive literature review that investigated the role of antioxidants in safeguarding Leydig cells against oxidative stress [96]. Two key defects in the steroidogenic pathway have been identified as contributing to age-related reductions in testosterone production: reduced LH-stimulated cAMP production and impaired cholesterol transport to and within the mitochondria.

Increasing oxidative stress appears to play a crucial role in age-related testosterone reduction, and aging is associated with enhanced lipid peroxidation. Leydig cell membranes from older rats exhibit a two- to three-fold increase in basal thiobarbituric acid-reactive substances (TBARSs) formation, indicating increased oxidative damage. They demonstrated that aging leads to a decrease in serum testosterone levels in both humans and rodents. This decline is not attributable to a loss of Leydig cells, but rather to their reduced capacity to produce testosterone in response to luteinizing hormone (LH) [97].

In spermatozoa, oxidative stress triggers lipid peroxidation of polyunsaturated fatty acids in sperm membranes [42,79], leading to protein aggregation in male germ cells, and potentially disrupting proteostasis [98]. Proteostasis in sperm cells is of pivotal significance for sustaining optimal functionality and fertility. It encompasses regulatory mechanisms that govern protein synthesis, folding, modification, and degradation [99]. Sperm cells are distinguished by their limited capacity for protein synthesis and repair, which renders proteostasis essential for their viability [99]. Disruptions in proteostasis can result in sperm dysfunction, impacting motility and fertilization potential; thus, the interplay between proteostasis and oxidative stress in sperm cells is pivotal to their functionality [98,99]. Proteostasis mechanisms have been shown to mitigate the harmful effects of oxidative stress [100]. Indeed, it has been demonstrated that the sustained production of misfolded proteins can exceed the capacity of the proteostasis network, resulting in its failure and subsequent cell death [101]. Morphological abnormalities in sperm are also frequently observed in men with high oxidative stress levels. These abnormalities, which may include defects in the acrosome, tail, or head, are often a result of lipid peroxidation and protein damage induced by ROS. These structural changes compromise the sperm’s ability to penetrate the egg and perform its fertilization function.

Male infertility can be influenced by various endogenous sources of ROS in seminal plasma. The human semen sample contains a variety of cells, including immature and mature spermatozoa, round-shaped cells of different phases of spermatogenesis, epithelial cells, and leukocytes [102]. A significant contributor is leukocytes, particularly polymorphonuclear leukocytes and macrophages, which originate from the seminal vesicles and prostate gland. In the presence of urogenital infections or inflammation, these leukocytes exhibit an enhanced immune response, generating up to 100 times more ROS, thereby leading to oxidative stress [103], while immature, morphologically abnormal spermatozoa also serve as primary sources of ROS [59]. However, the rate of ROS production is up to 1000 times higher in leukocytes (extrinsic source) compared to spermatozoa (intrinsic source) [104].

## 6. Spermatogenesis and Physical Activity: The Role of Oxidative Stress Control

Regular moderate-intensity physical exercise is associated with an increase in antioxidant defense systems and a reduction in systemic oxidative stress [105]. This is largely due to the adaptation of various enzymatic and non-enzymatic antioxidants, such as superoxide dismutase (SOD), catalase (CAT), and glutathione peroxidase (GPx), which play key roles in neutralizing ROS [106]. Furthermore, PA has been found to improve mitochondrial function, which may contribute to a more efficient regulation of oxidative metabolism and reduced ROS production [107]. Conversely, intense or prolonged PA, especially when performed in an unconditioned state, can result in an acute increase in oxidative stress. This phenomenon is primarily attributable to the enhanced production of ROS during high-intensity exercise, which can exceed the body’s antioxidant defense mechanisms [108,109].

As a result, oxidative damage can occur, leading to muscle fatigue, inflammation, and, potentially, impaired sperm quality. It is important to note that the beneficial effects of physical activity on oxidative stress are dose-dependent, with moderate levels of exercise being most effective in improving antioxidant capacity and mitigating the negative effects of oxidative stress [105,110].

### Impact of Physical Activity on Spermatogenesis

The etiology of male infertility is multifactorial, with physiological factors such as age and lifestyle factors, including physical activity, playing a role. However, the specific impact of sporting activity on semen parameters, and, consequently, on male fertility, remains unclear. Recent studies have explored the impact of physical activity on male fertility, highlighting the complex relationship between exercise and reproductive health [111,112,113,114].

**Figure 3:**
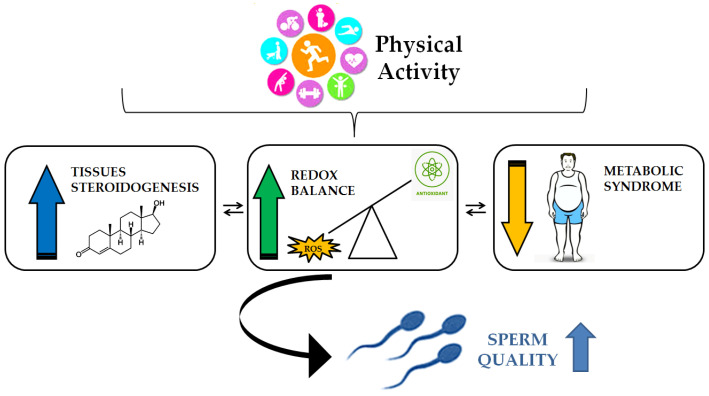
Physical activity can improve sperm quality by acting on various aspects of an individual’s health. It increases steroidogenesis (in muscles and in the testes), improves redox balance, enhances antioxidants, and reduces metabolic dysfunction. In addition, these factors interact and influence each other to improve sperm quality.

Moderate exercise has been shown to improve sperm quality, including count, motility, and morphology, while excessive or severe exercise may have detrimental effects [10,115,116]. Regular physical exercise has been shown to enhance sperm count, motility, and morphology in both human and rat subjects, with a concomitant improvement in testosterone, LH, and FSH levels in rats. PA has been associated with increased serum testosterone levels due to improved circulation and enhanced nutrient delivery to the testes. Moreover, acute and chronic exercise stimulate sex steroidogenesis enzymes expression and activity and sex steroid hormone levels in skeletal muscle tissue [117,118]. Exercise has been demonstrated to have a positive impact on the secretion of FSH, thereby enhancing the support and nourishment of developing sperm cells [119], leading to a higher sperm count and improved sperm quality [120]. It has been demonstrated that exercise can reduce the percentages of sperm with negative tubular differentiation (TDI) and spermiogenesis indices (SPIs) and DNA fragmentation, and can ameliorate diabetes-induced apoptosis and improve sperm apoptosis index in animal models [121]. The positive effects of exercise on male fertility are thought to be mediated by reduced oxidative stress, enhanced antioxidant defense, and improved steroidogenesis [115,116,117]. Furthermore, the improvement of glucose metabolism that results from an increase in physical activity is often the primary factor that ameliorates sperm parameters in men, since most studies performed involved men affected by metabolic syndrome and type 2 diabetes [117,122,123,124,125,126] (Figure 3).

Steroidogenesis, the process of producing steroids such as testosterone, plays a crucial role in regulating various physiological functions, including muscle growth, sexual function, and overall health. In this context, PA has been identified as a potential modulator of steroidogenesis, influencing both testicular and muscular pathways. Acute bouts of intense exercise, such as heavy resistance training or endurance exercise, lead to transient increases in testosterone secretion [127]. This response is thought to be mediated by the hypothalamic–pituitary–gonadal axis, which stimulates the release of LH and, consequently, the production of testosterone by Leydig cells [127]. Research suggests that the impact of physical activity on male fertility depends on the intensity and duration of the exercise, as well as the profile of the participant [121]. Studies involving recreational athletes demonstrated positive effects of prolonged physical activity. In fact, 433 infertile men training at 70–85% of their maximal oxygen consumption revealed that high-intensity exercise may restrain inflammatory biomarkers, oxidative stress, and antioxidants, while concomitantly enhancing semen parameters and the pregnancy rate [115,121].

While moderate exercise may have beneficial effects on sperm quality, high-intensity or prolonged exercise can negatively affect semen parameters [120,128,129]. Intense physical activity has been associated with decreased sperm concentration, motility, and morphology, particularly in elite athletes [129]. It has been observed that endurance exercise, including activities such as long-distance running or extensive cycling, has the potential to adversely affect seminal parameters [112]. A 16-week low-to-intensive cycling training program resulted in a decline in sperm quality parameters and an increase in seminal inflammatory markers, with some effects persisting even after a 30-day recovery period [130]. The same training regimen has also been shown to result in increased oxidative stress and decreased antioxidant capacity in semen [130]. A further study comparing endurance cyclists to sedentary controls found significantly lower proportions of morphologically normal sperm in cyclists [131]. At the same time, moderate running has been shown to improve cardiovascular health, reduce oxidative stress, and enhance hormone regulation, all of which can potentially benefit male fertility. Moreover, the regulation of insulin sensitivity and the reduction in visceral fat, both outcomes of consistent running, are linked to improved hormonal profiles, including better testosterone levels. However, excessive endurance running or high-intensity training may have the opposite effect, leading to a decrease in sperm quality [129]. This decline in sperm quality is attributed to mechanical impact, gonadal overheating, wearing tight clothes, and increased oxidative stress [59,129]; however, these effects may be reversible with proper rest and recovery periods [112].

Ultimately, the relationship between exercise and male fertility remains complex, with some studies showing conflicting results and difficulties in quantifying physical activity [10]. Further evidence showed the influence of physical exercise on the endocrine system, particularly the hypothalamo–pituitary–adrenal (HPA) axis and stress hormones production, in turn influencing testosterone production. The intensity and duration of exercise have been demonstrated to modulate the HPA axis response, resulting in increased cortisol secretion [132]. While endurance training does not lead to permanent hypercortisolism, it has been observed to result in decreased tissue sensitivity to glucocorticoids [132].

The relationship between cortisol and testosterone during exercise is a complex one, too. Post-exercise, a significant negative correlation between cortisol and total testosterone has been observed, while there is a positive correlation with free testosterone [133]. Prolonged imbalances in cortisol and growth hormone secretion can be detrimental to health [134]. In cases of overtraining, the sympathetic/parasympathetic imbalance and neuroendocrine dysfunction hypotheses have been proposed to explain performance decrements and recovery issues [135]. It is therefore crucial to ensure proper exercise planning with sufficient recovery to prevent overtraining and maintain hormonal balance.

Exercise-induced oxidative stress can be counteracted by the consumption of nutritional strategies [136] and antioxidant-rich foods [137], which can further support the maintenance of sperm quality. Antioxidant supplementation has shown potential in improving sperm quality and fertility outcomes in some individuals [137]. Specific antioxidants such as selenium, zinc, omega-3 fatty acids, CoQ10, and carnitines have been positively associated with sperm quality [138]. However, excessive antioxidant use may be detrimental to sperm function, resulting in a paradoxical decline in sperm quality [138], which is similar to the effect seen with oxidative stress.

However, an exact cut-off for “excessive” has not been well defined in the existing literature. A balanced diet rich in natural antioxidants from fruits, vegetables, whole grains, legumes, and seeds is recommended as a safe and effective approach to meet antioxidant requirements in physically active individuals and athletes [139].

## 7. Conclusions and Future Perspectives

This review underscores the significant role of exercise in modulating male reproductive health, primarily through its impact on oxidative stress and inflammatory pathways. The findings suggest that engaging in moderate-intensity exercise over a prolonged period can effectively suppress pro-inflammatory cytokine production, improve antioxidant defense mechanisms, and enhance sperm DNA integrity. Such effects are particularly beneficial for individuals experiencing infertility, as oxidative stress is known to impair sperm quality and overall testicular function. High-Intensity Interval Training (HIIT) has also demonstrated potential in improving sperm characteristics in men with fertility issues, further supporting the notion that structured physical activity can be an effective intervention for reproductive health. The testicles are highly susceptible to oxidative stress due to their high rate of cell division, mitochondrial oxygen consumption, and abundance of unsaturated fatty acids. As a result, excessive production of reactive oxygen species (ROS) can disrupt spermatogenesis, impair steroidogenesis, and reduce sperm quality. Exercise exerts a dual effect on male reproductive function—while mild-to-moderate exercise improves testicular steroidogenesis, spermatogenesis, and sexual competence by increasing insulin sensitivity and regulating ROS production, excessive and prolonged exercise can have adverse effects by promoting oxidative stress and impairing testicular function. This highlights the importance of maintaining an optimal balance in exercise intensity and duration to support reproductive health.

Furthermore, research has demonstrated that exercise can improve fertility in men with lifestyle-induced conditions such as obesity and diabetes. By enhancing testicular antioxidant defense, reducing pro-inflammatory cytokine levels, and promoting steroidogenesis, exercise contributes to improved spermatogenesis and semen quality. However, the extent to which exercise benefits male fertility is influenced by multiple factors, including an individual’s overall health status, the type, intensity, and duration of exercise, and pre-existing metabolic or hormonal conditions. These factors should be carefully considered when prescribing exercise as a therapeutic strategy for male reproductive health.

Future research should focus on refining clinical guidelines that consider individual health status, exercise volume, intensity, and duration. Further clinical trials are required to validate the underlying mechanisms and establish exercise-based interventions for managing lifestyle-induced infertility. A more comprehensive understanding of the interaction between physical activity and male reproductive health will facilitate the development of evidence-based recommendations that optimize fertility outcomes.

## 8. Review Limitations

This review was designed primarily as a narrative synthesis for providing a flexible overview of the role of physical activity in counteracting oxidative stress-induced sperm alteration by improving redox homeostasis control. The intention was to address broad topics and integrate diverse literature from different fields without restrictive guidelines, so as to facilitate the reading and understanding of such research, especially for non-expert audiences. It is recognized that the selection of articles leaves room for potential bias and that the analysis might inadvertently overlook key studies. Nevertheless, an informal assessment of methodological quality was performed by examining the clarity of each study’s objectives, the description of experimental or clinical methods, and the relevance of the outcome measures.

## Data Availability

Not applicable.

## Abbreviations

The following abbreviations are used in this manuscript:

cAMP	Cyclic adenosine monophosphate.
CAT	Catalase.
CO	Carbon monoxide.
Cx43	Connexin-43.
FSH	Follicle-stimulating hormone.
GPx	Glutathione peroxidase.
GnRH	Gonadotropin-releasing hormone.
H_2_O_2_	Hydrogen peroxide.
H2	Hydrogen.
LCS	Leukocytospermia.
LH	Luteinizing hormone.
NH3	Ammonia.
PKA	Protein kinase A.
PA	Physical activity.
Prdx2	Peroxiredoxin 2.
PUFA	Polyunsaturated fatty acids.
RES	Reactive electrophile species.
RNS	Reactive nitrogen species.
RHS	Reactive halogen species.
REDOX	Oxidation–reduction.
ROS	Reactive oxygen species.
SSCs	Spermatogonial stem cells.
SOD	Superoxide dismutase.
SPIs	Spermiogenesis indices.
TBARS	Thiobarbituric acid-reactive substances.
TDI	Tubular differentiation.
